# Embryo development is impaired by sperm mitochondrial-derived ROS

**DOI:** 10.1186/s40659-024-00483-4

**Published:** 2024-01-30

**Authors:** Yentel Mateo-Otero, Marc Llavanera, Marc Torres-Garrido, Marc Yeste

**Affiliations:** 1 Biotechnology of Animal and Human Reproduction (TechnoSperm), Institute of Food and Agricultural Technology, University of Girona, Girona, ES-17003 Spain; 2 Unit of Cell Biology, Department of Biology, Faculty of Sciences, University of Girona, Girona, ES- 17003 Spain; 3 Catalan Institution for Research and Advanced Studies (ICREA), Barcelona, ES-08010 Spain

**Keywords:** Sperm metabolism, Mitochondrial respiration, electron transport chain, Cyanide, Oocyte fertilisation, Embryo development, Oxidative phosphorylation

## Abstract

**Background:**

Basal energetic metabolism in sperm, particularly oxidative phosphorylation, is known to condition not only their oocyte fertilising ability, but also the subsequent embryo development. While the molecular pathways underlying these events still need to be elucidated, reactive oxygen species (ROS) could have a relevant role. We, therefore, aimed to describe the mechanisms through which mitochondrial activity can influence the first stages of embryo development.

**Results:**

We first show that embryo development is tightly influenced by both intracellular ROS and mitochondrial activity. In addition, we depict that the inhibition of mitochondrial activity dramatically decreases intracellular ROS levels. Finally, we also demonstrate that the inhibition of mitochondrial respiration positively influences sperm DNA integrity, most likely because of the depletion of intracellular ROS formation.

**Conclusion:**

Collectively, the data presented in this work reveals that impairment of early embryo development may result from the accumulation of sperm DNA damage caused by mitochondrial-derived ROS.

**Supplementary Information:**

The online version contains supplementary material available at 10.1186/s40659-024-00483-4.

## Background

The energetic metabolic signature of mammalian sperm influences reproductive outcomes, both in terms of oocyte fertilisation and embryo development [1, 2]. Indeed, mitochondrial membrane potential (MMP), which indirectly reflects the cellular capacity to produce ATP by oxidative phosphorylation (OXPHOS) [3], has been found to positively influence oocyte fertilisation in humans [4–6]. In spite of this, a metabolomics approach carried out using the pig as an animal model found that metabolites linked to OXPHOS were related to suboptimal in vitro fertilisation (IVF) outcomes in terms of altered embryo development [2]. While the underlying mechanisms of these associations are yet to be uncovered, one could reasonably suggest that reactive oxygen species (ROS) are implicated.

Sperm mitochondria have been proposed as a major source of ROS [7], produced from the leakage of respiratory chain electrons [8]. Under physiological conditions, ROS are involved in the regulation of sperm function, participating in the modulation of sperm capacitation and acrosomal reaction and, even, oocyte fertilisation [9]. Overproduction of ROS, nonetheless, results in oxidative stress, which has extensively been described to have harmful effects for sperm including the induction of membrane lipid peroxidation and DNA damage, the reduction of sperm motility and the impairment of oocyte fertilisation [7, 9, 10]. Focusing on the latter, sperm DNA fragmentation has been widely reported to compromise in vivo and in vitro fertility outcomes in both humans [11, 12] and other mammals [13–16].

Considering the lack of studies unfolding the mechanisms by which sperm metabolism influences the reproductive success, the present work aimed to evaluate the impact of ROS resulting from mitochondrial activity on IVF outcomes, as well as their underlying mechanisms. To this end, the relationship between mitochondrial activity, intracellular ROS levels and IVF outcomes was explored. Next, Complex IV (also known as cytochrome C oxidase), the last enzyme of the mitochondrial respiratory chain [17], was pharmacologically inhibited to address whether variations in mitochondrial activity affect sperm DNA integrity. In this work, Complex IV was blocked with cyanide, which is known to prevent electrons from reaching molecular oxygen, inhibiting the production of superoxides (O_2_^●−^), the precursor of most other ROS [18], through the electron transport chain. This approach allowed us to extend our knowledge on how sperm metabolism, and particularly mitochondrial activity, may shape fertility potential and have subsequent repercussions on early embryo development. Here we show that mitochondrial activity negatively influences embryo development, most likely due to the induction of sperm DNA damage by ROS derived from oxidative metabolism.

## Methods

### Reagents

Unless stated otherwise, the reagents used in the present study were purchased from Sigma (Merck, Darmstadt, Germany).

### Animals

All semen samples were acquired from an artificial insemination centre (Gepork S.L.; Masies de Roda, Spain). Management of animals by this centre is in accordance with the ISO certification (ISO-9001:2008), the EU Directive 2010/63/EU for animal experimentation, the Catalan Animal Welfare Law, and the current regulation on Health and Biosafety issued by the Department of Agriculture, Livestock, Food and Fisheries, Regional Government of Catalonia, Spain. No Ethics Committee permission was required to conduct this work because ejaculates were purchased from the artificial insemination centre and, therefore, no animal was manipulated for the purpose of the present study.

Semen samples were collected from healthy, sexually mature Pietrain boars (1–3 years old) using the gloved-hand method. Next, ejaculates were diluted to a final concentration of 33 × 10^6^ sperm/mL using a commercial extender (Vitasem LD, Magapor S.L., Zaragoza, Spain), and stored at 17ºC for 24 h. Before being included, basic sperm quality parameters including sperm viability and motility were assessed to check semen samples met the standard minimum requirements (> 80% of viable sperm and > 70% of motile sperm).

Moreover, ovaries were retrieved from pre-pubertal gilts sacrificed for food purposes at a local abattoir (Frigorífics Costa Brava; Riudellots de la Selva, Girona, Spain).

### Experimental design

The present study was divided into three experiments in order: (i) to explore the relationship between mitochondrial activity, ROS production and IVF outcomes; (ii) to validate the effect of cyanide on sperm function, mitochondria and intracellular ROS levels; and (iii) to evaluate whether mitochondrial activity influences sperm DNA integrity. For the first experiment, thirty-three ejaculates (each from a separate boar; i.e., 33 boars) were split into two aliquots. The first one was intended to assess sperm function, which included MMP, intracellular levels of total ROS and O_2_^●−^, and oxygen consumption rate (OCR). The second aliquot was used to perform IVF. For the second experiment, a total of twelve pools (each one composed of three ejaculates from three separate boars; i.e. three boars per pool) were split into three aliquots containing either 5 mM or 10 mM of cyanide, or H_2_O as a vehicle control. After 1 h of incubation, sperm function (sperm viability, sperm motility, MMP, intracellular levels of total ROS and O_2_^●−^, and OCR) were assessed. The third experiment was also carried out using six ejaculate pools (each one composed of three ejaculates from separate boars; i.e. three boars per pool). Again, each pool was divided into three aliquots containing 0, 5, or 10 mM of cyanide. Sperm function (sperm viability, sperm motility, MMP and intracellular ROS levels) were assessed at three time points (0 h, 24 h, and 72 h). In addition, 30 µL of each treatment per time point were snap-frozen at -80ºC in order to determine sperm DNA integrity by the Comet assay. All incubations with cyanide were performed at 17ºC in order to evaluate basal metabolic activity in sperm.

### Evaluation of sperm motility

After pre-warming semen samples at 38ºC for 15 min, 3 µL was placed into a Leja20 counting chamber (Leja Products BV; Nieuw-Vennep, The Netherlands). Each sample was evaluated individually using a Olympus BX41 microscope (Olympus; Tokyo, Japan) with a negative phase contrast objective (Olympus 10 × 0.30 PLAN objective, Olympus), through a computer-assisted sperm analysis system (Integrated Sperm Analysis System, ISAS V1.0; Proiser S.L.; Valencia, Spain). At least 1,000 sperm were examined per sample. The percentage of motile sperm, defined as those with an average path velocity ≥ 10 μm/s, was recorded.

### Flow cytometry

Sperm viability, MMP, and intracellular levels of total ROS and O_2_^●−^ were determined using a CytoFLEX cytometer (Beckman Coulter; Brea, CA, USA). Subcellular debris and cell aggregates were excluded using forward and side scatter, and gain and flow rate remained constant throughout the experiment. A minimum of 10,000 sperm events per sample were examined. All fluorochromes were purchased from ThermoFisher Scientific (MA, USA).

Samples were adjusted to a final concentration of 4 × 10^6^ sperm/mL in 1× PBS before fluorochrome staining. Sperm viability was assessed following the protocol of Garner and Johnson [19], which uses SYBR-14 to stain sperm nuclei, and propidium iodide (PI) to label sperm with compromised plasma membrane integrity. Briefly, samples were incubated with SYBR-14 (final concentration: 32 nM) and PI (final concentration: 7.5 µM) at 38ºC in the dark for 15 min. Fluorescence of SYBR-14 was detected through the fluorescein isothiocyanate channel (FITC; 525/40), and that of PI using the PC5.5 channel (690/50). Both fluorochromes were excited with the 488-nm laser and no compensation was applied. The percentage of viable sperm corresponded to the SYBR-14^+^/PI^−^ population, after subtracting the percentage of debris particles (SYBR-14^−^/PI^−^) in the analysis.

The MMP was evaluated following the protocol set by Ortega-Ferrusola et al. [20], which uses 5,5’,6,6’-tetrachloro-1,1’,3,3’tetraethyl-benzimidazolylcarbocyanine iodide (JC-1). Briefly, sperm were incubated with JC-1 (final concentration: 750 nM) at 38ºC in the dark for 30 min. In sperm with high MMP, JC-1 aggregates and emits orange fluorescence, which was here collected through the PE (585/42) channel. On the contrary, JC-1 is found in its monomeric form in sperm with low MMP and emits green fluorescence, which was collected through the FITC channel (525/40). Both JC-1 aggregates and monomers were excited using the 488-nm laser. The MMP of each sample was evaluated by the ratio between the mean fluorescence intensities of JC-1 aggregates and JC-1 monomers.

Total ROS levels were determined using the CellROX™ Deep Red Reagent. CellROX™ Deep Red is a cell-permeant dye that emits red fluorescence when oxidised by ROS. Sperm were incubated with CellROX™ Deep Red (final concentration: 2 µM) at 38ºC in the dark for 30 min. Then, PI (final concentration: 7.5 µM) was added and further incubated for 5 min at the same conditions. CellROX™ Deep Red was excited with the 638-nm laser and detected through the APC channel (660/20), whereas PI was excited with the 488-nm laser and detected through the PC5.5 channel (690/50). The CellROX™ Deep Red fluorescence intensity of viable sperm with high levels of intracellular total ROS (CellROX™ Deep Red^+^/PI^−^) was recorded to assess total ROS levels.

Intracellular O_2_^●−^ levels were assessed following the protocol of Guthrie and Welch [21], which uses hydroethidine (HE), a molecule that is oxidized into ethidium (E^+^) in the presence of O_2_^●−^. Briefly, samples were incubated with HE (final concentration: 5 µM) and YO-PRO-1 (final concentration: 31.25 nM) at 38ºC in the dark for 20 min. Samples were excited with the 488-nm laser, and fluorescence emitted by E^+^ and YO-PRO-1 was collected through PE (585/42) and FITC (525/40) channels, respectively. The fluorescence intensity of E^+^ in viable sperm with high levels of intracellular O_2_^●−^ (E^+^/ YO-PRO-1^−^) was recorded to assess O_2_^●−^ levels in semen samples.

### Oxygen consumption rate (OCR)

The SensorDish® Reader system (PreSens Gmbh; Regensburg, Germany) was used to evaluate OCR in sperm samples. One mL from each sample was transferred onto an Oxodish® OD24 plate, and the dish was then sealed with Parafilm®. Negative controls were performed by transferring the same volume of the cell-free medium in order to measure the background concentration of O_2_. Samples were then incubated at 38ºC for 1 h, and the O_2_ concentration in each well was measured every 30 s. The background of O_2_ concentration in cell-free wells was subtracted from every sample. Data were normalised against the percentage of viable cells, and the OCR was subsequently calculated as µM O_2_/h×10^6^ viable sperm.

### Oocyte maturation, in vitro fertilisation, and embryo culture

Ovaries were transported to the laboratory in 0.9% NaCl supplemented with 70 µg/mL kanamycin at 38ºC. Cumulus-oocyte complexes (COC) were extracted from follicles and only those exhibiting a complete, compact cumulus mass were included in the experiment. Selection of COCs was carried out using Dulbecco’s PBS (Gibco, ThermoFisher) supplemented with 4 mg/mL BSA.

Oocytes were in vitro matured using TCM-199 (Gibco), supplemented with 0.57 mM cysteine, 0.1% (w:v) polyvinyl alcohol, 10 ng/mL human epidermal growth factor, 75 µg/mL penicillin-G potassium, and 50 µg/mL streptomycin sulphate. First, groups of 50–60 COCs were matured in four-well multi-dishes (Nunc, ThermoFisher; Waltham, MS, USA) containing 500 µL of pre-equilibrated maturation medium supplemented with 10 IU/mL equine chorionic gonadotropin (eCG; Folligon; Intervet International B.V.; Boxmeer, The Netherlands) and 10 IU/mL human chorionic gonadotropin (hCG; Veterin Corion; Divasa Farmavic S.A.; Gurb, Barcelona, Spain). After 22 h, oocytes were moved to 500 µL fresh pre-equilibrated maturation medium without hormones.

After mechanically removing cumulus cells, the matured oocytes were transferred into 50-µL drops of pre-equilibrated IVF medium (Tris-buffered medium [22]) containing 1 mM caffeine in groups of 20–30 oocytes. In parallel, semen samples were adjusted to 1,000 sperm per oocyte in IVF medium. Next, gametes were co-incubated at 38.5ºC and 5% CO_2_ for 5 h. A total of 100 oocytes per semen sample were inseminated. The putative zygotes were then moved onto 500 µL NCSU23 medium [23] supplemented with 0.4% BSA, 0.3 mM pyruvate, and 4.5 mM lactate for in vitro embryo culture. After two days, fertilisation rate was assessed by calculating the percentage of cleaved embryos, which were then transferred into NCSU23 medium supplemented with 0.4% BSA ad 5.5 mM glucose. Six days after fertilisation, the resulting embryos were classified following Balaban & Gardner [24] criteria. Specifically, percentages of morulae, early blastocysts/blastocysts, hatching/hatched blastocysts and total embryos (sum of morulae, early blastocysts/blastocysts and hatching/hatched blastocysts) were evaluated. In addition, two different ratios were calculated: (i) the developmental potential at day 6, which resulted from dividing the percentage of morulae, early blastocysts/blastocysts and hatched/hatching blastocysts by the percentage of 2–8 cell embryos; and (ii) the developmental competency of fertilised embryos, which was the number of embryos at day 6 divided by the number of embryos at day 2.

### Comet assay

For the evaluation of DNA integrity (single- and double-strand breaks), the Comet assay protocol set by Ribas-Maynou et al. [25] was followed. First, samples were diluted to 5 × 10^5^ sperm/mL, and mixed with 0.66% low melting point agarose (37ºC). Then, two 6.5-µL drops of the mixture were poured onto agarose pre-treated slides. Next, drops were covered with an 8-mm round coverslip and agarose was jellified at 4ºC for 5 min. After gently removing the coverslips, slides were incubated in three lysis solutions: (1) 0.8 M Tris-HCl, 0.8 M DTT and 1% SDS for 30 min; (2) 0.8 M Tris-HCl, 0.8 M DTT and 1% SDS for 30 min; and (3) 0.4 M Tris-HCl, 0.4 M DTT, 50 mM EDTA, 2 M NaCl, 1% Tween20 and 100 µg/mL Proteinase K for 180 min. Then, a denaturalisation step was performed in cold (4ºC) alkaline solution (0.03 M NaOH, 1 M NaCl) for 5 min before slides were electrophoresed in an alkaline buffer (0.03 M NaOH, pH = 13) at 1 V/cm for 4 min. Finally, the electrophoresed slides were incubated in neutralisation solution (0.4 M Tris-HCl, pH = 7.5) for 5 min, and dehydrated in an ethanol series (70%, 90% and 100%) for 2 min each. Sperm DNA was stained with 5 µL of 1× Safeview DNA stain (NBS biological, Huntingdon, UK), and covered with a coverslip.

Comets were observed and captured under an epifluorescence microscope (Zeiss Imager Z1; Carl Zeiss AG, Oberkochen, Germany). At least 100 sperm cells per sample were captured at 100× magnification, with a resolution of 1388 × 1040 pixels and using Axiovision 4.6 software (Carl Zeiss AG, Oberkochen, Germany). Capture time was constant for all samples.

Fluorescence intensities of Comet heads and tails were analysed through open-access CometScore v2.0 software (Rexhoover, www.rexhoover.com). Captures not corresponding to cells, overlapping comets, or those that showed impurities in the head or tail signal were manually removed. For the quantification of the amount of sperm DNA breaks, olive tail moment (OTM), calculated as (Tail mean intensity – Head mean intensity) × Tail DNA / 100, was chosen as a reference parameter.

### Statistical analyses

Statistical analyses were conducted with a statistical package (IBM SPSS for Windows Ver. 27.0; IBM Corp., Armonk, NY, USA). Plots were elaborated using GraphPad Prism 8.0 Software (GraphPad, San Diego, USA). Data was first tested for normality (Shapiro-Wilk test) and homogeneity of variances (Levene test). The level of significance was set at *P* ≤ 0.05.

For the first experiment, Pearson correlations between mitochondria-related parameters, total ROS and O_2_^●−^ levels, and IVF outcomes were calculated. In addition, sperm samples were classified by their in vitro fertility potential. A principal component analysis was run using the percentage of cleaved embryos at day 2, morulae, early blastocysts/blastocysts, hatching/hatched blastocysts, and total embryos (sum of morulae, early blastocysts/blastocysts and hatching/hatched blastocysts) at day 6. Varimax with Kaiser normalization was utilised as the rotation method. The analysis yielded two components explaining 77.1% of the total variance. Thereafter, a two-step cluster analysis was performed using regression factors of these two components to classify sperm samples on the basis of their in vitro fertility potential (measure of distance: log-likelihood; clustering criterion: Schwarz’s Bayesian Criterion, BIC), with the number of groups being determined automatically. Then, mitochondria-related parameters were compared between the two groups through a t-test for independent samples. In addition to this, sperm samples in the first experiment were also clustered considering OCR, total ROS and developmental potential through a two-step cluster analysis (measure of distance: log-likelihood; clustering criterion: BIC) to compare the OCR, ROS and embryo development potential between sperm samples.

For experiments 2 and 3, data of each biological replicate were first normalised to their control within each time point. Then, one-way ANOVA for each timepoint was performed, followed by Dunnett’s post-hoc test. For Comet analysis, OTM values of each spermatozoon were used to run a two-step cluster analysis (measure of distance: log-likelihood; clustering criterion; BIC), with the number of groups being determined automatically. This resulted in two subpopulations of sperm (high and low sperm DNA fragmentation), the cut-off value being 27.80. Following this, the proportions of each sperm subpopulation in every sample were calculated. Data were standardised by calculating the ratio between the proportion of each sperm subpopulation at a given time point (0 h, 24 h, 72 h) and treatment (control, 5 mM, 10 mM cyanide) with respect to the control at that time point. The resulting ratios were then used to run a linear mixed model (repeated measures) where the inter-subject factor was cyanide concentration, and the intra-subject factor was the time of incubation. Pair-wise comparisons were made with Bonferroni’s test.

## Results

### Sperm mitochondrial activity and intracellular ROS have a negative impact on IVF outcomes

**Fig. 1:**
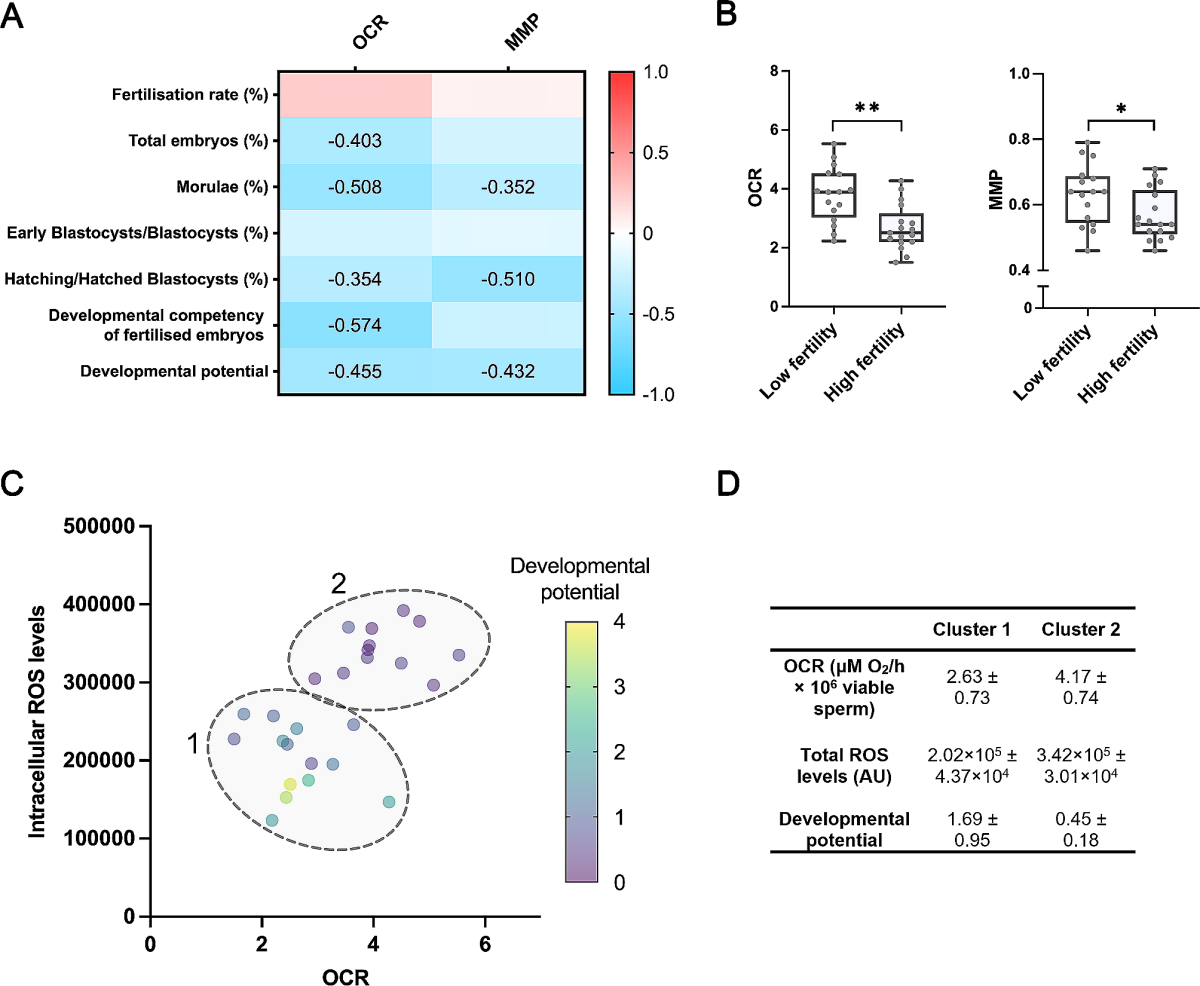
Association of mitochondrial activity with ROS levels and in vitro fertilisation outcomes. (**A**) Correlation heatmap between mitochondrial activity parameters and in vitro fertility outcomes (*n* = 33). The colour saturation from red to blue represents Pearson correlation coefficients (R) from 1 to -1, respectively. Only significant correlations (*P* < 0.05) are numerically indicated. (**B**) Comparison of OCR (µM O_2_/h·10^6^ viable sperm) and MMP (ratio) of samples classified as with low (*n* = 16) or high (*n* = 17) in vitro fertility potential. Data are represented as mean ± standard error of the mean. Significant differences (*P* < 0.05 and *P* < 0.01) are marked with * and **, respectively. (**C**) Sample clustering (*P* < 0.05) considering total ROS levels (AU), OCR (µM O_2_/h·10^6^ viable sperm) and embryo development potential (ratio). Each dot represents a biological replicate (*n* = 26), and their colouring depends on embryo development potential. (**D**) Descriptive values of each parameter (OCR (µM O_2_/h·10^6^ viable sperm), total ROS levels (AU) and developmental potential (ratio)) of the two clusters (*P* < 0.05). Data are represented as mean ± standard error of the mean. OCR: oxygen consumption rate. MMP: mitochondrial membrane potential. ROS: reactive oxygen species

Mitochondrial activity was here evaluated using two distinct approaches, OCR and MMP. Significant Pearson correlations (*P* < 0.05) are shown in Fig. 1A. No relationship between any of these two parameters and fertilisation rate was found (*P* > 0.05). On the other hand, OCR was found to be negatively correlated (*P* < 0.05) to the percentage of embryos at day 6 (*R* = -0.403), the percentage of morulae (*R* = -0.508), and the percentage of hatched/hatching blastocysts (*R* = -0.354). In addition, a negative correlation (*P* < 0.05) between OCR and both developmental competency of fertilised embryos (*R* = -0.574) and developmental potential of morulae (*R* = -0.455) was observed. Regarding MMP, a negative correlation (*P* < 0.05) was seen with the percentage of morulae (*R* = -0.352) and the percentage of hatching/hatched blastocysts (*R* = -0.510). Moreover, MMP was also observed to be negatively correlated to developmental potential of morulae (*R* = -0.432).

Following these observations, samples were divided into two groups depending on their in vitro fertility potential: high and low in vitro fertility potential. Next, OCR and MMP were compared between these two groups (Fig. 1B), finding lower levels of both (*P* < 0.05) in samples classified as high fertility compared to those classified as low fertility (OCR, high fertility group: 2.68 ± 0.77 µM O_2_/h×10^6^ viable sperm vs. low fertility group: 3.80 ± 0.94 µM O_2_/h×10^6^ viable sperm; MMP, high fertility group: 0.57 ± 0.07 vs. low fertility group: 0.63 ± 0.09).

Next, the relationship between mitochondrial activity and ROS production was also assessed. Total ROS levels were found to be positively correlated (*P* < 0.05) to both OCR (*R* = 0.682) and MMP (*R* = 0.414). Conversely, no correlation between intracellular O_2_^●−^ levels and mitochondrial activity parameters was found.

After confirming the association between total ROS and mitochondrial activity, the relationship of each of these parameters with in vitro fertility outcomes was explored (Fig. 1C) and two distinct clusters were identified (*P* < 0.05). Remarkably, the poorest embryo development resulted from semen samples exhibiting high levels of both OCR and total ROS (Fig. 1D).

### Cyanide inhibits sperm mitochondrial activity

**Fig. 2:**
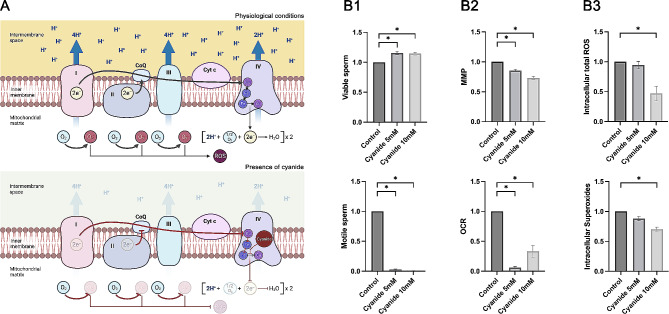
Effect of cyanide on mitochondrial-derived ROS. (**A**) Schematic representation of the mechanisms by which cyanide disrupts the mitochondrial electron transport chain. Under physiological conditions, respiratory complexes I, II and III leak electrons to oxygen producing the superoxide anion (O_2_^●−^), which can be later converted into reactive oxygen species (ROS) [18]. In the presence of cyanide, the heme a_3_-Cu_B_ binuclear centre of Complex IV is blocked [32], thus preventing electrons from reaching molecular oxygen and inhibiting the electron transport chain [18]. Not only does this inhibition prevent the formation of O_2_^●−^ and derived ROS products along the electron transport chain, but it also reduces MMP and OCR. (**B**) Effect of cyanide (5 mM and 10 mM) on sperm viability, sperm motility, MMP, OCR, and intracellular levels of total ROS and superoxides (O_2_^●−^). Cyanide treatments were normalised to the control in each biological replicate (*n* = 12). Data are represented as mean ± standard error of the mean. Significant differences (*P* < 0.05) are marked with *. OCR: oxygen consumption rate. MMP: mitochondrial membrane potential. ROS: reactive oxygen species

To further explore the molecular mechanisms through which OXPHOS in sperm could compromise embryo development, mitochondrial activity was inhibited using cyanide. In this second experiment, effects on cyanide on sperm were evaluated by incubation with two different concentrations (5 mM and 10 mM) for 1 h. Each biological replicate was normalised to its control (0 mM, cyanide vehicle). The effect of cyanide on mitochondrial-derived ROS is schematically represented in Fig. 2A. This figure represents both the data extracted from the literature and the results obtained herein.

First, the impact of cyanide on sperm viability and motility was assessed (Fig. 2B1). The two concentrations of cyanide were found to dramatically decrease (*P* < 0.01) the percentage of motile sperm compared to the control (0.03 ± 0.03 and 0.01 ± 0.01 vs. 1.00 ± 0.00, respectively). On the other hand, the percentage of viable sperm in treatments containing 5 mM and 10 mM cyanide was significantly higher (*P* < 0.01) than in the control (1.15 ± 0.09 and 1.15 ± 0.08 vs. 1.00 ± 0.00, respectively).

In addition, cyanide was observed to successfully inhibit sperm mitochondrial activity at the two concentrations tested (Fig. 2B2). In effect, both 5 mM and 10 mM cyanide were detected to reduce MMP and OCR (*P* < 0.01) compared to the control (for OCR: 0.06 ± 0.33 and 0.06 ± 0.36 vs. 1.00 ± 0.00; for MMP: 0.86 ± 0.07 and 0.73 ± 0.11 vs. 1.00 ± 0.00, respectively).

Finally, the effect of mitochondrial inhibition on intracellular ROS levels was interrogated (Fig. 2B3). Interestingly, after 1 h of incubation, only the treatment containing 10 mM cyanide showed significantly lower levels (*P* < 0.01) of total ROS compared to the control (0.47 ± 0.42 vs. 1.00 ± 0.00, respectively). In addition, intracellular O_2_^●−^ levels in the two cyanide treatments (5 mM and 10 mM) were lower (*P* < 0.01) than in the control (0.88 ± 0.12 and 0.70 ± 0.13 vs. 1.00 ± 0.00, respectively).

### Inhibition of mitochondrial activity reduces intracellular ROS levels and sperm DNA damage

In order to explore if the ROS resulting from OXPHOS had any effect on sperm DNA integrity, semen samples were incubated with 5 mM and 10 mM cyanide for 72 h. Each biological replicate was normalised to its control (0 mM, cyanide vehicle).

**Fig. 3:**
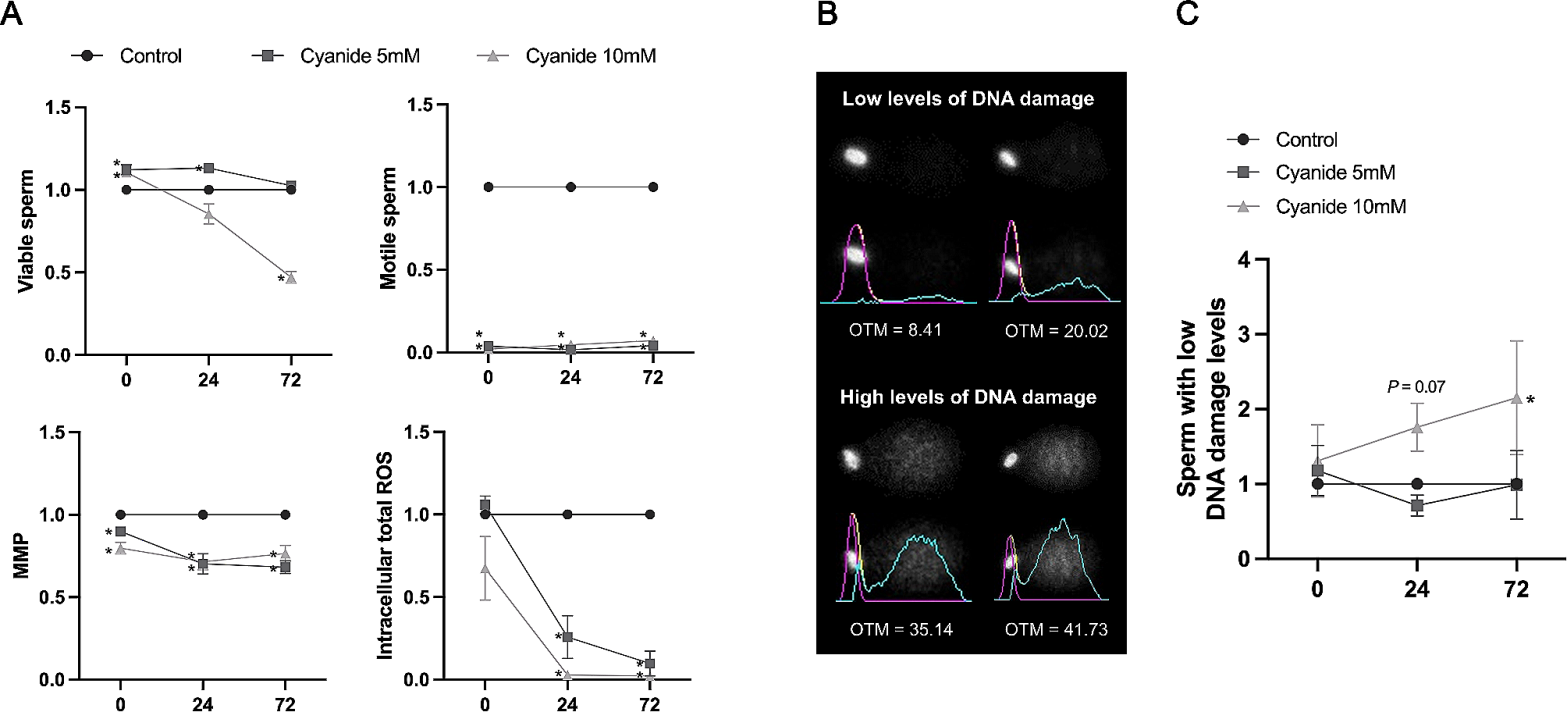
Effect of mitochondrial activity on sperm DNA integrity. (**A**) Effect of cyanide (5 mM and 10 mM) on sperm viability, sperm motility, MMP and total ROS levels after 72 h of incubation. Cyanide treatments were normalised to the control of each biological replicate (*n* = 6). Data are represented as mean ± standard error of the mean. Significant differences (*P* < 0.05) are marked with *. (**B**) Representative images for the two clusters (*P* < 0.05), sperm with low and high levels of DNA damage. The lower images represent Comet analysis using the Cometscore v2 software. The cyan line indicates the tail area, the magenta line indicates the head area, and the yellow line indicates the total area. (**C**) Effect of 5 mM and 10 mM cyanide on sperm DNA damage after 72 h of incubation. The graph shows the impact of cyanide treatments on the subpopulation of sperm with low DNA incidence. Cyanide treatments were normalised to the control of each biological replicate (*n* = 6). Data are represented as mean ± standard error of the mean. Significant differences (*P* < 0.05) are marked with *. OCR: oxygen consumption rate. MMP: mitochondrial membrane potential. OTM: olive tail moment. ROS: reactive oxygen species

First, the effect of cyanide over time was evaluated (Fig. 3A). As observed before, the two cyanide treatments were found to compromise (*P* < 0.01) the percentage of motile sperm throughout the entire incubation period. Regarding sperm viability, at 0 h, the percentage of viable sperm was greater (*P* < 0.05) in both 5 mM and 10 mM cyanide-treated samples than in the control (1.12 ± 0.07 and 1.11 ± 0.63 vs. 1.00 ± 0.00, respectively). This pattern was maintained after 24 h of incubation in the case of 5 mM cyanide compared to the control (1.13 ± 0.06 vs. 1.00 ± 0.00, respectively; *P* < 0.01). Furthermore, after 72 h of incubation, the percentage of viable sperm in samples treated with 10 mM cyanide was lower (*P* < 0.01) than in the control (0.47 ± 0.09 vs. 1.00 ± 0.00, respectively). In addition, MMP in samples incubated with 5 mM or 10 mM cyanide was lower (*P* < 0.01) than in the control over the entire incubation period (at 0 h: 0.90 ± 0.06 and 0.80 ± 0.09 vs. 1.00 ± 0.00; at 24 h: 0.70 ± 0.15 and 0.71 ± 0.12 vs. 1.00 ± 0.00; at 72 h: 0.68 ± 0.10 and 0.77 ± 0.12 vs. 1.00 ± 0.00, respectively). Finally, total ROS levels were noticeably lower (*P* < 0.01) in samples incubated with 5 mM or 10 mM cyanide after 24 and 72 h of incubation compared to the control (at 24 h: 0.26 ± 0.31 and 0.03 ± 0.01 vs. 1.00 ± 0.00; at 72 h: 0.10 ± 0.18 and 0.02 ± 0.01 vs. 1.00 ± 0.00, respectively).

Finally, the impact of mitochondrial respiratory chain inhibition on sperm DNA integrity was also explored in the present work (Fig. 3B). The individual OTM values of each spermatozoon were used to cluster cells, and two sperm subpopulations resulted from this analysis: low (from 0.00 to 27.79) and high (from 27.80 to 59.38) incidence of sperm DNA damage. Next, the proportions of each sperm subpopulation in every sample were determined and, for each biological replicate, the ratio between the proportion of each sperm subpopulation in each cyanide treatment with respect to the control was calculated for all time points. Focusing on the subpopulation of sperm with low DNA damage, no significant differences between treatments and the control were observed at the beginning of the experiment (Fig. 3C). Yet, in presence of 10 mM cyanide, the proportion of sperm with low DNA damage levels increased over time, being significantly higher (*P* < 0.05) than the control after 72 h of incubation (2.01 ± 3.87).

## Discussion

Mitochondrial activity has been extensively reported to be relevant for oocyte fertilisation in mammals [2, 4–6, 26], possibly because of its involvement in motility regulation during capacitation [3] and acrosome reaction [27, 28]. In spite of this, a recent metabolomics approach carried out in porcine sperm reported that higher levels of metabolites related to OXPHOS were associated to poorer embryo development [2]. To further investigate these associations, the relationship between two OXPHOS indicators (MMP and OCR) and IVF outcomes was here addressed. Interestingly, and in accordance with previous results [2], while none of these two mitochondria-related parameters were found to be associated with fertilisation rate, high levels of both variables were observed to negatively impact embryo development evaluated at day 6. Considering that mitochondrial activity, and therefore OXPHOS, is known to inherently trigger the generation of ROS [29], we hypothesised that these associations could be caused by the adverse effects of ROS resulting from energetic metabolism on non-capacitated sperm. Indeed, the association between mitochondrial activity and intracellular ROS was evaluated, evidencing an obvious and positive association between them. Remarkably, our results also revealed that sperm samples resulting in low embryo development potential exhibited high levels of MMP and ROS. Although it has been suggested that high amounts of ROS may impair mitochondrial function potentially causing sperm dysfunction and, consequently, infertility [30], this would not be in accordance with the findings observed herein. For this reason, we speculated that a more feasible explanation for such a decreased developmental potential could involve sperm DNA integrity.

To test this hypothesis, the specific effects of mitochondrial activity on sperm were evaluated by the disruption of the last enzyme of the electron transport chain, i.e., Complex IV. Complex IV catalyses the transfer of electrons from cytochrome C to molecular oxygen in order to produce H_2_O [17]. Under physiological conditions, Complex IV translocates protons from the mitochondrial matrix to the intermembrane space, thus contributing to the formation of an electrochemical gradient that ultimately allows ATP generation [31]. Here we inhibited this complex using cyanide, a specific inhibitor of the heme a_3_-Cu_B_ binuclear centre of Complex IV [32]. Cyanide binding to Complex IV prevents electrons from reaching molecular oxygen, thus blocking the electron transport chain [18]. As, to the best of our knowledge, this was the first time that this molecule was used in mammalian sperm for this purpose, we initially tested the effects of incubating sperm with cyanide for 1 h. Data showed that cyanide dramatically decreases MMP and OCR after 1 h of incubation and, therefore, efficiently interrupts mitochondrial activity. We also observed that this incubation did not compromise sperm viability, but disrupted sperm motility. The fact that sperm motility was completely abolished in the presence of cyanide proves that OXPHOS is crucial for the regulation of sperm motility. This would be in agreement with previous research in humans, horses, rodents and pigs, where mitochondrial activity was found to play a key role in sustaining motility [27]. Still related to this matter, another relevant finding from our experiment was that disrupting mitochondrial respiration was observed to drastically reduce intracellular ROS levels, particularly O_2_^●−^ production. This is likely to be a consequence of the inhibition of other complexes of the electron transport chain, which have been reported to be the main source of ROS [18]. In any case, this reduction could also potentially explain the enhanced sperm survival observed in samples treated with cyanide.

On the other hand, our data provides some clues about the origin of intracellular ROS under basal conditions. Intracellular ROS in sperm have been proposed to have two main origins: (i) mitochondria and (ii) plasma membrane [33]. In more detail, recent studies suggested sperm oxidases residing in the plasma membrane, mainly NADPH oxidases (NOX), as a main source of ROS during mammalian sperm capacitation [33, 34]. A different situation, however, seems to occur under non-capacitating conditions. In effect, our results support that, in non-capacitating conditions, most of the ROS within the cell are originated from mitochondrial metabolism. These findings open the possibility of further characterising the mechanisms regulating ROS production in sperm under different physiological conditions, particularly the main origin of these ROS (i.e., plasma membrane and mitochondria) as well as their potential implications for these cells.

Having established the intimate association between sperm OXPHOS and ROS production, we last aimed to examine whether the ROS originated from oxidative metabolism could have an impact on sperm DNA integrity. Our data show that the inhibition of mitochondrial activity, and thus the decrease of intracellular ROS levels, results in decreased sperm DNA damage compared to basal conditions. These results would support the hypothesis that sperm samples with increased OXPHOS exhibit suboptimal IVF outcomes because of the induction of DNA damage [2], which has been previously described to affect early mammalian embryo development [11, 14, 16]. It is worth mentioning that our results would seem contradictory with the data from previous studies in humans, in which: (i) semen samples with low MMP were found to have increased sperm DNA damage [28], and (ii) sperm with active mitochondria showed lower sperm chromatin damage [6]. These apparent inconsistences could be explained by the intrinsic sperm quality of semen samples. Specifically, the two studies conducted in humans explored the relationship between subpopulations of sperm with distinct mitochondrial activity and sperm quality variables, one of those being sperm DNA integrity. For this reason, while these two studies in humans addressed, through an observational approach, whether mitochondrial activity relies upon the initial sperm quality – and, with this purpose, samples of different sperm quality were tested –, our work took an experimental angle to investigate the repercussion of mitochondrial activity on sperm DNA integrity. In any case, several mechanisms have already been proposed to explain how ROS might compromise DNA integrity: (i) OH^●−^ radicals directly generate oxidised DNA adducts, destabilising the DNA structure and causing single-strand breaks; (ii) ROS indirectly activates endonucleases that cause DNA breaks; and, (iii) during cellular apoptosis, ROS stimulate caspases that further allow the release of mitochondria free radicals [35]. The exact molecular pathways by which DNA breaks are induced in sperm need to be further explored in future studies.

## Conclusions

**Fig. 4:**
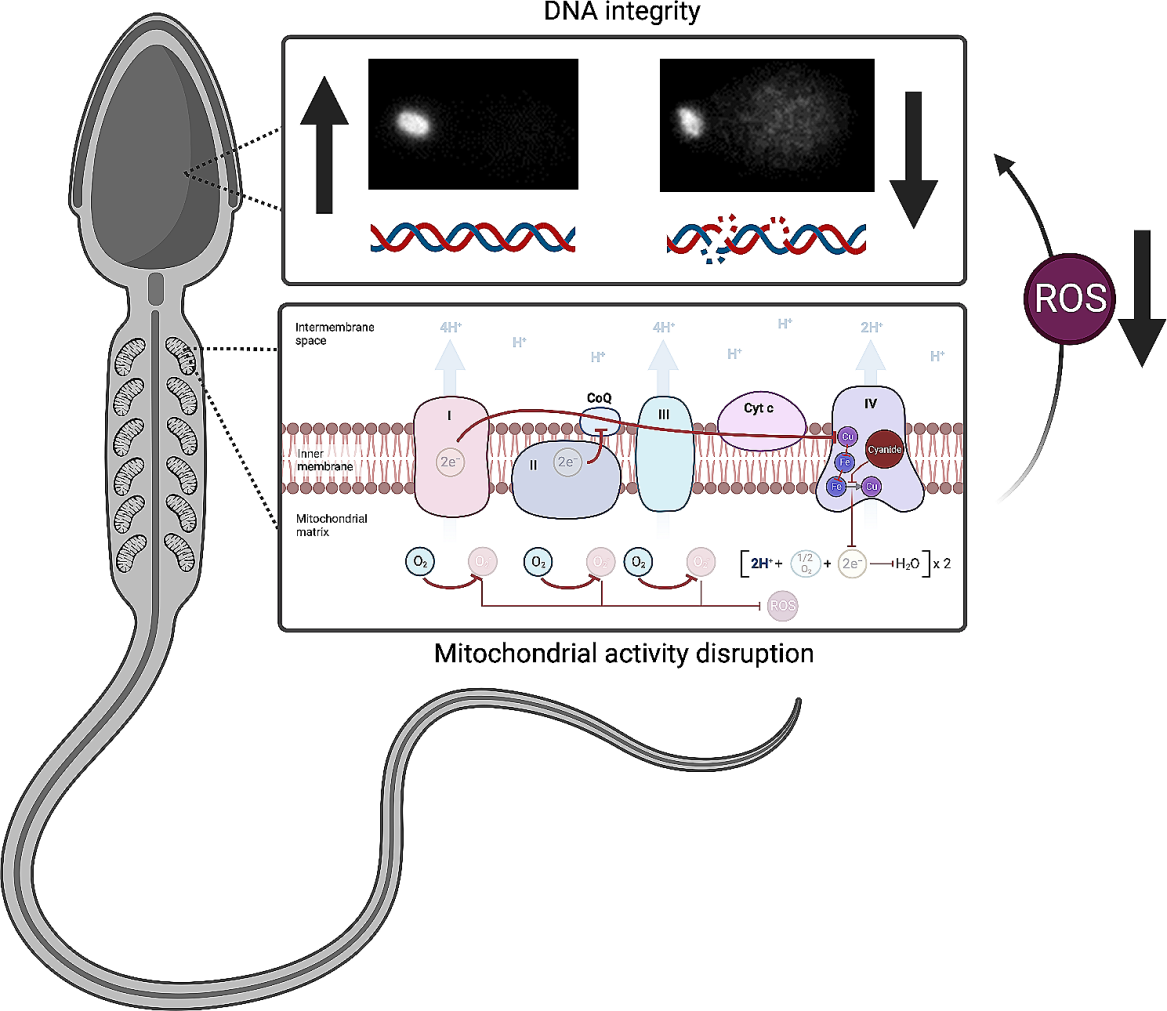
Schematic representation of the main results of the present work. Mitochondrial activity inhibition by cyanide drastically drops intracellular ROS levels in sperm. This decrease possibly prevents the induction of further DNA damage. ROS: reactive oxygen species

The present work underscores the influence of mitochondrial activity on sperm function and fertility potential, not only encompassing its consequent impact on subsequent embryo development but also the underlying physiological causes. First, we showed that most of the intracellular ROS in sperm is originated from mitochondrial activity. In effect, we propose that the ROS resulting from OXPHOS in non-capacitating sperm are able to influence early embryo development through the induction of sperm DNA damage (Fig. 4). In addition, and consistently to previous studies [5, 6, 36, 37], the results presented herein support the notion that the determination of sperm mitochondrial activity could be used as a potential predictor for the success of assisted reproductive technology.

### Electronic supplementary material

Below is the link to the electronic supplementary material.

Supplementary Material 1

## Data Availability

Data underlying the findings described in the present manuscript are available as supporting information.

## Abbreviations

COC: Cumulus-oocyte complexes
HE: Hydroethidine
IVF: In vitro fertilisation
JC-1: 5,5’,6,6’-tetrachloro-1,1’,3,3’tetraethyl-benzimidazolylcarbocyanine iodide
MMP: Mitochondrial membrane potential
O_2_^●−^: Superoxide
OCR: Oxygen consumption rates
OTM: Olive-tail moment
OXPHOS: Oxidative phosphorylation
PI: Propidium iodide
ROS: Reactive oxygen species
